# Surgical Treatment of Neovascular Glaucoma Secondary to Proliferative Diabetic Retinopathy in Japanese Patients without the Use of Glaucoma Drainage Devices

**DOI:** 10.3390/jcm13113252

**Published:** 2024-05-31

**Authors:** Masaru Takeuchi, Takayuki Kanda, Kozo Harimoto, Daisuke Sora, Rina Okazawa, Tomohito Sato

**Affiliations:** Department of Ophthalmology, National Defense Medical College, Tokorozawa 359-8513, Japan; kankan@ndmc.ac.jp (T.K.); fwgi0461@ndmc.ac.jp (K.H.); doc37044@ndmc.ac.jp (D.S.); doc36008@ndmc.ac.jp (R.O.); dr21043@ndmc.ac.jp (T.S.)

**Keywords:** diabetic retinopathy, neovascular glaucoma, trabeculectomy, diabetes, vitrectomy

## Abstract

**Purpose:** The purpose of this study is to investigate outcomes of visual acuity (VA) and intraocular pressure (IOP) in proliferative diabetic retinopathy (PDR)-associated neovascular glaucoma (NVG) in Japanese patients treated with surgical therapies without the use of glaucoma drainage devices. **Methods:** A retrospective analysis of medical records was conducted for 31 consecutive PDR-associated NVG patients who underwent surgical treatments in our institution between 2013 and 2022. Patient demographics, clinical characteristics, VA, and IOP were recorded at the first and last visits, and surgical procedures, including pars plana vitrectomy with extensive panretinal and ciliary photocoagulation (PPV–PRCP), diode laser trans-scleral cyclophotocoagulation (DCPC), and trabeculectomy with mitomycin C (TLE–MMC), with or without a prior intravitreal bevacizumab (IVB) injection, were reviewed. **Results:** Of the thirty-one PDR patients with NVG, two patients received PPV–PRCP or DCPC alone (6.5%), respectively, three patients received TLE–MMC alone (9.7%), two patients received TLE–MMC after IVB (6.5%), six patients received PPV–PRCP and TLE–MMC (19.4%), seven patients received PPV–PRCP and TLE–MMC after IVB (22.6%), five patients received PPV–PRCP and DCPC and TLE–MMC (16.1%), and four patients received PPV–PRCP and DCPC and TLE–MMC after IVB (12.9%). The VA of two patients (6.5%) deteriorated to no light perception. In all patients, the mean logMAR VA was 1.28 ± 1.05 at the first visit and remained at 1.26 ± 1.08 at the last visit, with no significant change; the mean IOP was 33.0 ± 15.2 mmHg at the initial visit and decreased significantly to 14.0 ± 7.4 mmHg at the last visit. The number of eyes with IOP ≥ 21 decreased from twenty-eight (90.3%) to three (9.7%). Although IOP in patients with IOP > 30 mmHg at the initial visit reduced to a level comparable to that of patients with IOP ≤ 30 mmHg, the IOP > 30 mmHg group received IVB more frequently and had significantly higher logMAR VA at the last visit compared to the IOP ≤ 30 mmHg group. Hypotony (<6 mmHg) was observed in four eyes (12.9%). **Conclusions:** In PDR patients with NVG, various combinations of PPV–PRCP, DCPC, and TLE–MMC after adjunctive IVB without the use of glaucoma drainage devices lowered IOP sufficiently; for these patients, neovascular regression was observed, with no further deterioration of VA. However, surgical procedures should be performed for PDR patients with NVG before visual impairment occurs. On the other hand, approximately less than 15% of patients developed blindness or low IOP.

## 1. Introduction

Neovascular glaucoma (NVG) secondary to proliferative diabetic retinopathy (PDR) is a devastating disease that can cause blindness, continuous pain, and eventually the loss of the eye [1]. Retinal ischemic conditions trigger the release of angiogenic growth factors such as vascular endothelial growth factor (VEGF), hypoxia-inducible factor 1-alpha (HIF1a), and angiopoietin-2, which drive neovascular growth in the iris and fibrovascular tissue proliferation in the anterior chamber angle, obstructing the trabecular meshwork and causing elevated intraocular pressure (IOP) [2]. In addition, a confluent fibrovascular membrane leads to extensive peripheral anterior synechiae (PAS) formation with a subsequent further rise in IOP and the exacerbation of glaucomatous damage [3]. The management of NVG associated with DR involves both reducing the underlying ischemia with further panretinal photocoagulation (PRP) [4] and lowering IOP with pharmacotherapy and/or surgery. 

A large number of studies have confirmed that PRP significantly improves the ischemic condition of the retina and promotes the regression of neovascularization [4,5]. However, PRP alone may not be fully effective for PDR patients who have developed NVG [6]. Pars plana vitrectomy (PPV) is the main treatment modality for PDR, which involves removing intraocular angiogenic factors. Wide-angle viewing systems, offering a panoramic view of the surgical field, improve the safety and efficacy of vitreoretinal surgical procedures. Bimanual maneuvers using endolasers and scleral compression under chandelier illumination and wide-angle viewing systems allow the photocoagulation of the ciliary processes beyond the pars plana, which promotes the regression of neovascularization [7]. This procedure is considered to be comparable to endoscopic cyclophotocoagulation, which uses an endoscope combined with a diode laser [8].

However, if the fibrovascular membrane in the anterior chamber angle leads to PAS formation and the elevated IOP cannot be controlled with anti-glaucoma eye drops, surgical intervention is required to lower the IOP. Cyclophotocoagulation uses high-intensity lasers to irradiate the ciliary epithelium and stroma to reduce aqueous humor production, resulting in lowering of the IOP [9,10,11]. In addition, cyclophotocoagulation is considered to reduce the oxygen demand by the ciliary tissues, which should compensate for the incomplete effect of PRP to ameliorate retinal ischemia.

Trabeculectomy (TLE) with mitomycin C (MMC) is the most common filtration surgery to reduce IOP, but is less efficient for cases of NVG in which scar formation has progressed; in these cases, post-surgery complications are unavoidable. Bevacizumab is a neutralizing anti-VEGF monoclonal antibody that binds to all forms of VEGF-A. When injected into the vitreous or anterior chamber, bevacizumab causes the rapid regression of neovascularization in the anterior segment, leading to a decrease in IOP [12,13,14,15,16,17,18,19,20,21,22]. Several studies have demonstrated that a preoperative intravitreal injection of bevacizumab (IVB) inhibits hemorrhagic complications associated with TLE in NVG patients and improves the relatively poor surgical outcome [23,24,25,26]. Glaucoma drainage devices (GDDs), including valved and non-valved implants, have been shown to effectively lower intraocular pressure (IOP) in patients with neovascular glaucoma (NVG) [27,28,29,30]. However, these devices are also associated with serious postoperative complications, including corneal edema, drainage tube exposure, endophthalmitis, intraocular hemorrhage, choroidal detachment, and vitreous hemorrhage [31].

In this study, we investigated the outcomes of visual acuity (VA) and IOP in Japanese patients with PDR-associated NVG who were treated with surgical therapies, such as PPV with extensive panretinal and ciliary photocoagulation (PRCP), diode laser trans-scleral cyclophotocoagulation (DCPC), and TLE with MMC after adjunctive intravitreal bevacizumab injection (IVB), without the use of glaucoma drainage devices. 

## 2. Materials and Methods

### 2.1. Patients

This is a retrospective, single-center cohort study. The clinical charts of 31 consecutive patients who were diagnosed with neovascular glaucoma (NVG) associated with proliferative diabetic retinopathy (PDR) and treated with surgical procedures at the National Defense Medical College Hospital between April 2011 and March 2022 were reviewed. PDR was diagnosed in cases of the presence of microaneurysms, intraretinal hemorrhages, cotton wool spot, venous beading, intraretinal microvascular abnormalities, and the neovascularization of the optic disc or elsewhere observed using fundus photography, and in cases of microaneurysms, retinal capillary non-perfusion, vascular telangiectasia, capillary drop outs, and neovascularization observed using fluorescein angiography, before or after surgical procedures [32]. NVG was defined as the presence of neovascularization in the anterior chamber angle or iris [33]. This study included PDR patients with NVG whose IOP was controlled below 21 mmHg using anti-glaucoma eye drops, but those whose IOP was below 21 mmHg without anti-glaucoma eye drops were excluded. This study adhered to the tenets of the Declaration of Helsinki and was reviewed and approved by the ethics committee of the National Defense Medical College Hospital (No. 4972). Consent of participation was obtained by an opt-out method with descriptions of the study protocol posted on the hospital website, and written informed consent was waived by the ethics committee due to the retrospective clinical chart review design of the study. For DR patients in whom NVG developed in both eyes, the right eye was evaluated in this study. Patients with corneal disease that caused visual impairment, primary glaucoma, retinal disease other than DR, uveitis, optic nerve disease, or a history of trauma or glaucoma surgery were excluded. 

### 2.2. Surgical Techniques

The regression of neovascularization in the anterior segment was generally attempted by performing procedures in the order of conventional PRP, PPV with PRCP, and then DCPC. For eyes with IOP higher than 21 mmHg refractory to medical treatment, TLE with MMC after adjunctive IVB was performed after the sufficient regression of neovascularization in the anterior segment was achieved. The use of IVB before TLE was decided upon by the operating ophthalmologist. All procedures were performed by experienced surgeons.

#### 2.2.1. Pars Plana Vitrectomy with Extensive Panretinal and Ciliary Photocoagulation

When neovascularization in the anterior segment was not regressed by conventional PRP alone, a three-port PPV was performed with a 25- or 27-gauge cutter. PPV in phakic eyes was performed after the completion of cataract surgery. Triamcinolone acetonide was used to facilitate the visualization of the vitreous, and posterior vitreous detachment was induced if it had not yet occurred. PRCP (1300–1800 shots) was performed up to 1/2 to 2/3 circumference of the ciliary process.

#### 2.2.2. Diode Laser Trans-Scleral Cyclophotocoagulation 

Patients were anesthetized with a retrobulbar injection of 2 mL of 2% lidocaine; under anesthesia, the footplate of the G-probe (OcuLight SLx, IRIS Medical Instruments, Mountain View, CA, USA) was placed approximately 0.5 mm behind the limbus, and irradiation was performed using a continuous-wave semiconductor diode laser at a wavelength of 810 nm. In almost all cases, 270° of the circumference was treated, and either the supero-temporal or superior sector (site of previous surgeries) was spared. The pulse duration was fixed at 2 s. The pulse power and the number of applications varied from case to case and among the surgeons. In general, the pulse power was set at 1750 or 2000 mW initially, and was either maintained throughout the procedure or adjusted according to the audible “pops”. Postoperatively, an antibiotic/steroid ointment was instilled.

#### 2.2.3. Intravitreal Bevacizumab Injection

In Japan, insurance does not cover anti-VEGF therapy for PDR with NVG, and bevacizumab is the only anti-VEGF agent approved for off-label use by the ethics committee of the National Defense Medical College. Therefore, bevacizumab was used for intravitreal injections in PDR patients with NVG from whom informed consent was obtained for this treatment. After topical anesthesia with 0.4% oxybuprocaine, the eyelid and eye were sterilized with povidone iodine. An eyelid speculum was inserted to stabilize the eyelid, and 1.25 mg/0.05 mL of bevacizumab (Avastin^®^; Roche Pharmaceuticals, Basel, Switzerland) was injected intravitreally through the pars plana. After injection, antibiotics were administered.

#### 2.2.4. Trabeculectomy with Mitomycin C 

After the creation of a fornix-based conjunctival flap, a half-thickness scleral flap of 3 × 3 mm was created. Small pieces of surgical sponge soaked in 0.5 mg/mL MMC were inserted under the conjunctival flap for 2 to 3 min. The eye was then irrigated thoroughly with 100 mL of saline. Trabeculectomy was performed with a Kelly Descemet’s Membrane Punch (Inami, Tokyo, Japan), followed by peripheral iridectomy. The scleral and conjunctival flaps were sutured with 10–0 nylon. Postoperatively, argon laser suture lysis and the needling revision of the filtration bleb were performed as necessary to enhance filtration.

### 2.3. Outcomes and Statistical Analysis

We investigated demographic and systemic data (sex, age, fasting blood glucose, HbA1c, creatinine, blood urea nitrogen, and estimated glomerular filtration rate), ocular findings (best-corrected VA and IOP), history of PRP, cataract surgery, and PPV at the initial visit, surgical procedures received (cataract surgery, PPV with PRCP, DCPC, IVB, and TLE with MMC), follow-up periods from the last surgery, and ocular outcome (VA and IOP) at the last visit. For the statistical analysis, VA was calculated as 0.0025 for counting fingers, 0.002 for hand motion, 0.0016 for light perception, and 0.0013 for no light perception [34].

Data of continuous variables are expressed as mean ± standard deviation or median and range. The Mann–Whitney U-test and Pearson’s chi-square test were used to compare the data between the two groups. All the statistical analyses were performed using JMP Pro ver.15.0 statistical software for Macintosh (SPSS, Chicago, IL, USA).

## 3. Results

### 3.1. Background and Treatment History of DR Patients with NVG

Table 1 shows the background and treatment history at the initial presentation of PDR patients with NVG enrolled in this study. The mean age was 64.2 ± 12.0 years and the male to female ratio was 22:9. As systemic factors, fasting blood glucose was 147.9 ± 38.8 mg/dL, HbA1c was 7.4 ± 1.5%, creatinine was 2.1 ± 3.0 mg/dL, blood urea nitrogen was 26.7 ± 16.6 mg/dL, and the estimated glomerular filtration rate was 55.1 ± 27.7 mL/min/1.73 m^2^. Regarding ocular findings, the mean log minimum angular resolution VA (logMAR VA) was 1.28 ± 1.05, and IOP was 33.0 ± 15.2 mmHg. Regarding the treatment history in ophthalmology, anti-glaucoma eye drops were prescribed in all eyes, PRP was performed in 25 eyes (80.6%), cataract surgery was performed in 23 eyes (74.2%), and PPV was performed in 18 eyes (58.1%). No patients had a history of TLE or DCPC.

### 3.2. Surgical Procedures and the Numbers Performed for NVG in DR Patients

**Figure 1:**
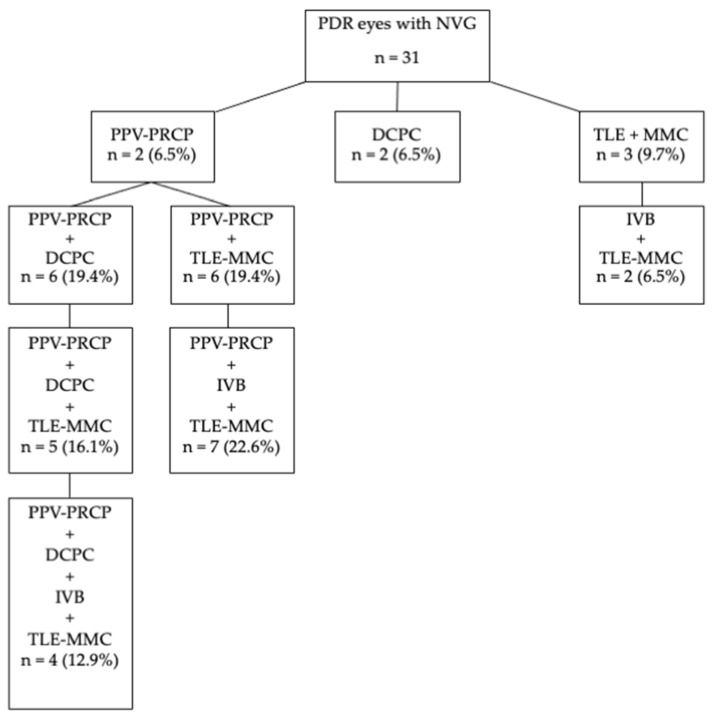
Surgical procedures performed for diabetic retinopathy eyes with neovascular glaucoma. PPV = pars plana vitrectomy, PRCP = panretinal and ciliary photocoagulation, DCPC = diode laser cyclophotocoagulation, IVB = intravitreal bevacizumab injection, TLE = trabeculectomy, MMC = mitomycin C.

Figure 1 shows various surgical treatments including various combinations performed on PDR eyes with NVG. PPV with PRCP (PPV–PRCP) or DCPC alone was performed in 6.5% of cases, TLE with MMC (TLE–MMC) was performed in 9.7% of cases, TLE–MMC after IVB was performed in 6.5% of cases, PPV–PRCP + DCPC or TLE–MMC was performed in 19.4% of cases, PPV–PRCP + DCPC + TLE–MMC was performed in 16.1% of cases, PPV–PRCP + TLE–MMC after IVB was performed in 22.6% of cases, and PPV–PRCP + DCPC + TLE–MMC after IVB was performed in 12.9% of cases. 

Table 2 summarizes the numbers of individual surgical procedures performed for each PDR eye with NVG. The most frequent procedure was TLE-MMC (87.1%, mean 1.2 times per eye), followed by PPV-PRCP (77.4%, mean 1.5 times per eye), IVB (41.9%, mean 0.5 times per eye), and DCPC (35.5%, mean 0.4 times per eye). 

### 3.3. Effects of Surgical Procedures on VA and IOP in DR Patients with NVG

**Figure 2:**
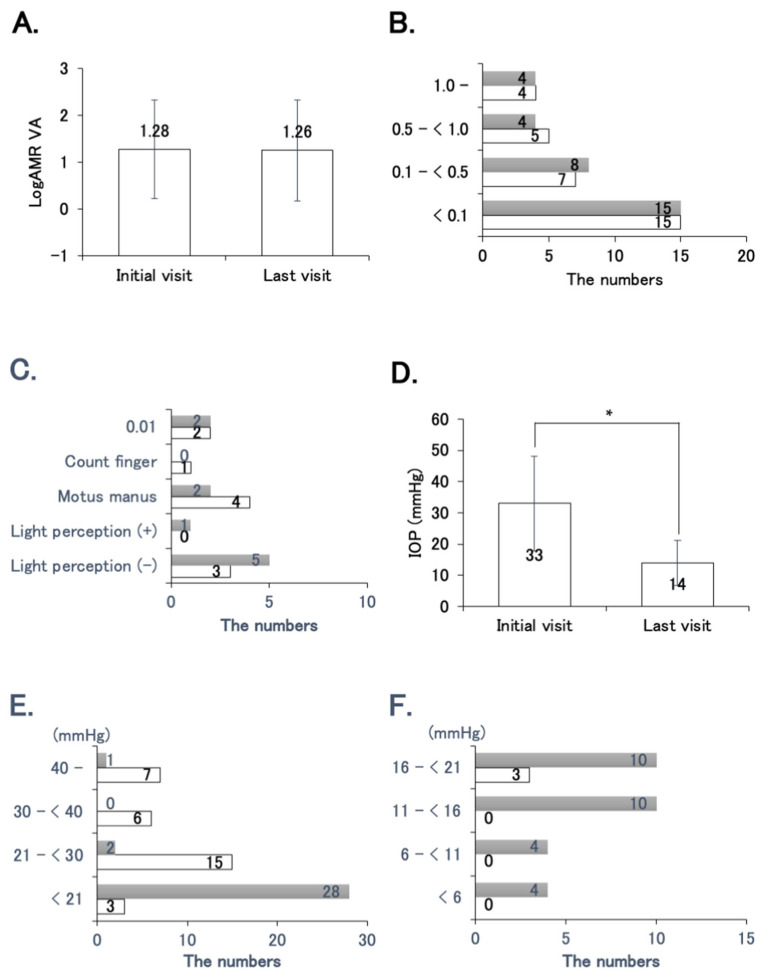
Visual acuity (VA) and intraocular pressure (IOP) in patients with neovascular glaucoma secondary to proliferative diabetic retinopathy at the initial and the last visits. (**A**) Mean logMAR VA at the initial and the last visits. (**B**) Numbers of eyes with best corrected visual acuity (BCVA) < 0.1, 0.1 to <0.5, 0.5 to <1.0, and ≥1.0 at the first visit (white bars) and the last visit (gray bars). (**C**) Numbers of eyes with BCVA 0.01, counting fingers, hand motion, light perception (+), and light perception (−) at the first visit (white bars) and the last visit (gray bars). (**D**) Mean IOP at the initial and the last visits. (**E**) Numbers of eyes with IOP < 21, 21 to <30, 30 to <40, and ≥40 at the first visit (white bars) and the last visit (gray bars). (**F**) Numbers of eyes with IOP < 6, 6 to <11, 11 to <16, and 16 to <21 at the first visit (white bars) and the last visit (gray bars). * *p* < 0.0001.

Figure 2 shows VA and IOP at the initial and the last visit. The mean logMAR VA at the first visit was 1.28 ± 1.05 and that at the last visit was 1.26 ± 1.08 (Figure 2A), with no significant difference between the two visits. The distribution of BCVA was similar at both the initial and the last visit, and almost one-half of the eyes were less than 0.1 (Figure 2B). However, when comparing eyes with BCVA less than 0.1, light perception (+) increased from zero at the initial visit to one eye (3.2%) at the last visit and light perception (−) increased from three eyes (9.7%) to five eyes (16.1%) (Figure 2C). The mean IOP was 33.0 ± 15.2 mmHg at the initial visit and decreased significantly to 14.0 ± 7.4 mmHg at the last visit (Figure 2D). The number of eyes with IOP ≥ 21 decreased from twenty-eight eyes (90.3%) at the initial visit to three eyes (9.7%) at the last visit; and eyes with IOP less than 21 mmHg increased from three eyes (9.7%) to twenty-eight eyes (90.3%) (Figure 2E). However, when comparing eyes with IOP less than 21 mmHg, hypotony (<6 mmHg) was observed in four eyes (12.9%) at the last visit (Figure 2F). 

### 3.4. Comparison of TLE–MMC with Prior IVB and without IVB

When NVG patients who received TLE–MMC after IVB were compared to those without IVB (Table 3), the IVB (+) group was younger (62.6 ± 10.4 vs. 67.1 ± 12.9) and had higher IOP (35.2 ± 12.1 mmHg vs. 33.9 ± 18.8 mmHg) at the initial visit. At the last visit, although logMAR VA appeared to be higher in the IVB (+) group than in the IVB (−) group (1.44 ± 1.06 vs. 1.19 ± 1.06), IOP tended to be lower in the IVB (+) group (11.4 ± 4.8 mmHg) than in the IVB (−) group (15.9 ± 9.1 mmHg) and IOP higher than 21 mmHg was not observed in the IVB (+) group. 

### 3.5. Comparison between logMAR VA above 1 and 1 or below at Initial Visit

When NVG patients with logMAR VA > 1 at the initial visit were compared to those with logMAR VA of 1 or below (Table 4), the group with logMAR VA > 1 had a significantly higher proportion of females and higher IOP (40.5 ± 18.4 mmHg vs. 26.0 ± 5.9 mmHg). This group also tended to be younger on average, had a lower history of PRP, cataract surgery, and PPV, and had higher levels of FBS and HbA1c than the group with logMAR VA ≤ 1. At the last visit, logMAR VA was still significantly higher in the group with logMAR VA > 1 than those in the logMAR VA ≤ 1 group (1.86 ± 1.01 vs. 0.70 ± 0.82). There was no difference in IOP between the two groups. The surgical procedures performed were also not significantly different between the two groups.

### 3.6. Comparison between IOP above 30 mmHg and 30 mmHg or below at Initial Visit

When NVG patients with IOP higher than 30 mmHg at the initial visit were compared to those with IOP of 30 mmHg or lower (Table 5), the group with IOP higher than 30 mmHg tended to be younger on average, had a higher logMAR VA (1.62 ± 1.04 vs. 1.03 ± 1.02), and a lower history of PRP than those with IOP of 30 mmHg or lower. At the final visit, the IOP of the group with IOP > 30 mmHg had been reduced to a level comparable to that of the IOP ≤ 30 mmHg group. However, IVB was performed more frequently (0.8 ± 0.7 vs. 0.3 ± 0.5) and logMAR VA was significantly higher in the IOP > 30 mmHg group than in the IOP ≤ 30 mmHg group (1.81 ± 1.03 vs. 0.87 ± 0.96).

## 4. Discussion

NVG secondary to PDR is a refractory disease that requires surgical intervention, as it cannot be controlled with medical therapy alone. It is associated with a risk of blindness. The mean IOP of 33.0 ± 15.2 mmHg at the first visit decreased significantly to 14.0 ± 7.4 mmHg at the last visit, and the number of eyes with IOP ≥ 21 decreased from twenty-eight eyes (90.3%) to three eyes (9.7%). In the three eyes with IOP exceeding 21 mmHg at the final visit, iris rubeosis showed minimal improvement, and two of these eyes already presented with light perception (−) at the initial visit. Ultimately, light perception did not remain in five out of the thirty-one studied eyes, including the aforementioned three eyes. This suggests that surgical intervention may not have been pursued aggressively in these eyes, and/or the optimal timing for therapy might have been missed.

The diabetic retinopathy study emphasizes that PRP reduces the risk of severe visual disturbance in patients with high-risk PDR or severe non-PDR by 50% to 60% [35]. However, standard PRP is sometimes insufficient to treat neovascular deterioration when neovascularization has already developed in the anterior segment in DR eyes. Except for one NVG eye in which IOP was reduced to the normal range using conventional PRP and anti-glaucoma eye drops, our first-line surgical treatment for NVG secondary to PDR was PPV with PRCP under chandelier illumination and wide-angle viewing systems. The photocoagulation of the ciliary process was adjusted subjectively according to the preoperative IOP and apparent activity of iris rubeosis. Therefore, when the concomitant use of photocoagulation to the ciliary process was inadequate, iris neovascularization was sometimes exacerbated after the first PPV with PRCP. In such cases, this procedure was repeated in an attempt to regress the iris neovascularization before other surgical procedures were considered. Consequently, PPV with PRCP was performed 1 to 4 times per NVG eye, with an average of 1.4 times in all the patients. 

The destruction of the ciliary body by inhibiting aqueous humor production to reduce IOP was first described in 1930 by Vogt [36]. Although the main complications of cyclodestruction include prolonged ocular inflammation, hypotony, phthisis bulbi, visual loss, postoperative pain, and intraocular hemorrhage [37], the use of a diode laser emits a beam with a wavelength of 800–850 nm, which is best absorbed by the melanin in the pigmentary epithelium, reducing the energy that affects the sclera [10]. DCPC may offer a more sophisticated and safer method over other forms of cyclodestruction and is one of the accepted options as an effective treatment for lowering IOP and relieving pain in advanced cases of NVG [2,9]. Successful IOP control rates of DCPC for NVG eyes have been reported to be 60% at 2 years [10] and 50–56% at 3 years [38]. In this study, although DCPC was performed in combination with other surgical procedures, the successful IOP control rate in NVG eyes that underwent DCPC was nine of eleven (81.8%) eyes for a mean follow-up period of 40.4 ± 37.2 weeks. In contrast, Ilive et al. [9] reported that among 131 eyes treated with DCPC, IOP decreased from 36.9 mmHg to 15.3 mmHg with a success rate of 69.5%, but hypotony (lower than 6 mmHg) occurred in 17.6%. Rami et al. [39] also reported hypotony (lower than 5 mmHg) as the most important risk of DCPC for NVG eyes, which was observed in 39% of the patients studied. In the present study, hypotony (lower than 6 mmHg) was found in four eyes (12.9%) at the last visit, which was an apparently lower rate compared with previous reports. The primary reason for this result is that we used DCPC for neovascular regression rather than for lowering IOP via suppressing aqueous humor production with cyclodestruction. If the regression of rubeosis was achieved, no further DCPC was applied, even when IOP was still high, and IOP was adjusted using subsequent TLE with MMC. In fact, there was no significant difference in IOP between eyes with and without DCPC; conversely, IOP was higher in the former. Fong et al. [37] compared DCPC alone and DCPC with IVB for NVG patients and reported that DCPC alone was effective in lowering IOP in NVG patients, and the addition of IVB did not statistically enhance treatment outcomes. On the other hand, Strzalkowski et al. performed CPC combined with IVB, PRP, and vitrectomy for 77 eyes with NVG, and achieved effective IOP reduction, visual acuity preservation, and the reduction of antiglaucoma medications, with phthisis bulbi as the only late complication in 3.9% of eyes [40]. We also used IVB in our study but for a different purpose; IVB was employed to improve outcomes of TLE–MMC in our series. Of the eleven eyes that underwent DCPC, nine eyes received IVB and two eyes did not. Although the number is quite small, the additional use of IVB did not result in the further lowering of IOP, which is similar to a previous report [37]. 

TLE–MMC after adjunctive treatment with IVB and PRP was reported to be a better treatment modality in the management of patients with NVG [6,41]. PRP is the most important factor that reduces neovascularization in the anterior segment and the use of IVB delays the need for glaucoma surgery [12,13,14,15,16,17,18,19,20,21,22]. IVB prior to TLE with MMC decreases bleeding during surgery and postoperative hyphema, which results in better IOP reduction [23,25]. Alkawas et al. [6] reported that IVB and PRP achieved the complete regression of iris neovascularization in 82.4% of the patients, and IOP was reduced from 42.9 ± 4.2 mmHg to 16.3 ± 2.0 and 19.7 ± 2.1 mmHg at the first and sixth month, respectively, with subsequent TLE–MMC. Also, in the present study, the median IOP was 11 mmHg in NVG patients who received TLE–MMC after IVB and 16 mmHg in those without IVB, and IOP higher than 21 mmHg was not observed in NVG patients who underwent TLE with MMC after IVB. However, further investigation is needed to evaluate the long-term efficacy and safety of IVB in managing NVG. In fact, one study found no significant long-term improvement in TLE with MMC outcomes with IVB [24]. On the other hand, for NVG patients in whom high IOP persisted and was not controlled with anti-glaucoma eye drops, ophthalmologists were reluctant to perform TLE–MMC after IVB before the adequate regression of iris neovascularization was achieved with repeated PPV with PRCP and DCPC. Most of these cases required multiple PPVs with PRCP and TLEs with MMC.

GDDs including valved and non-valved implants are recommended for NVG because of their high efficiency and safety in reducing IOP [27,28,29,30]; however, we do not offer these procedures due to the serious postoperative complications such as corneal edema, exposure of the drainage tube, endophthalmitis, hyphema, choroidal detachment, and vitreous hemorrhage [31]. In addition, in the comparison of TLE after intravitreal ranibizumab (IVR) with Ahmed glaucoma valve (AGV) implantation for NVG eyes, it has been reported that TLE with IVR offers the same or a better IOP-lowering effect [42,43]. Tokumo et al. [24] also reported similar results in IOP reduction, VA, and the number of medications between the TLE and Baerveldt implantation performed for NVG patients, and that the TLE group showed less late complications and better visual outcomes. Regarding cyclophotocoagulation, a literature review and meta-analysis has indicated that there appears to be no difference in the IOP-lowering efficacy between GDDs and cyclophotocoagulation for NVG patients, although GDDs appear to be safer [44].

In this study, two patients (6.5%) lost light perception after surgical procedures. Nevertheless, in all the subjects studied, logMAR VA at the initial visit and at the last visit did not change significantly, and the distribution of BCVA also showed little changes. However, compared to the group with lower logMAR VA (1 or less), the group with higher logMAR VA (greater than 1) at baseline had a significantly higher proportion of females and higher IOP, tended to be younger, less likely to have received prior PRP and PPV, and had higher blood sugar levels. These results suggest that younger and female DR patients did not receive adequate treatment until VA deteriorated further compared with older and male DR patients. At the final visit, although the group with higher logMAR VA and that with lower logMAR at baseline showed an IOP decrease to a similar extent, logMAR VA was still significantly higher in the group with higher logMAR VA at baseline. Likewise, compared with patients with lower IOP (30 mmHg or lower), NVG patients with higher IOP (higher than 30 mmHg) at baseline were significantly younger and appeared to have higher logMAR VA and less likely to have received prior PRP at the initial visit. At the last visit, although IOP was lowered to comparable levels in the two groups, IVB was used significantly more often and logMAR VA was still higher in the group with higher IOP at baseline. It is possible that irreversible visual impairment has already progressed in those eyes, indicating the importance of the early diagnosis and early treatment of NVG before visual impairment progresses.

The surgical procedures performed for PDR patients with NVG were diverse, either alone or in combination (Figure 1). Therefore, the number of cases in this study was not sufficient to statistically compare VA and IOP outcomes between the surgical procedures performed, and the reliability of the results was considered to be low even if it was performed. We are undertaking to investigate the effective surgical treatments and the combinations with a larger number of cases in a multicenter study.

We are aware of limitations of the present study, such as the retrospective design, single facility, use of only Japanese participants, uncertain duration from DR onset to the initial visit, difference in severity of PDR and NVG among patients, selection of surgical procedures and timing of procedures decided by individual ophthalmologists, and different follow-up periods. Although all surgical procedures were performed under similar conditions and data collection was relatively complete, some surgeons could have failed to enter all the procedural details. 

## 5. Conclusions

In patients with NVG secondary to PDR, employing various combinations of PPV with PRCP, DCPC, and TLE with MMC after adjunctive IVB without the use of glaucoma drainage devices significantly reduced neovascularization and decreased the proportion of patients with high IOP from 90.3% to 9.7%, without further visual acuity loss. Performing TLE with MMC after adjunctive IVB was more effective in lowering IOP compared to performing TLE with MMC alone. Although the surgical treatments for NVG patients with IOP > 30 mmHg at the initial visit lowered IOP to comparable extent as patients with IOP ≤ 30 mmHg at the last visit, the worse visual acuity was not improved, suggesting that surgical procedures should be performed for PDR patients with NVG before visual impairment occurs. Hypotony was induced in four patients (12.9%), and two patients lost light perception (6.5%). Further studies are warranted to assess the long-term outcomes and complications associated with these surgical interventions.

## Figures and Tables

**Table 1:** Background of proliferative diabetic retinopathy patients with neovascular glaucoma enrolled in this study at the initial visit.

	DR Patients with NVG (*n* = 31)
Age (years)	64.2 ± 12.0 * (63, 41–85) ^†^
Male/Female	22/9
Follow-up periods (weeks)	34.8 ± 36.0
Clinical factors	
FBS (mg/dL)	147.9 ± 38.8 (147, 82–255)
HbA1c (%)	7.4 ± 1.5 (7.6, 5.0–12.6)
Creatinine (mg/dL)	2.1 ± 3.0 (1.0, 0.5–12.7)
BUN (mg/dL)	26.7 ± 16.6 (21.5, 10–80)
eGFR (mL/min/1.73 m^2^)	55.1 ± 27.7 (59, 3.9–105.5)
Ocular conditions	
LogMAR VA	1.28 ± 1.05 (1.0, 0–2.89)
IOP (mmHg)	33.0 ± 15.2 (28.3, 16.5–78)
Treatment history in ophthalmology	
Anti-glaucoma eye drops	31 (100) ^§^
PRP	25 (80.6)
Cataract surgery	23 (74.2)
PPV	18 (58.1)
DCPC	0 (0)
TLE	0 (0)

DR = diabetic retinopathy, NVG = neovascular glaucoma, FBS = fasting blood sugar, BUN = blood urea nitrogen, eGFR = estimated glomerular filtration rate, LogMAR VA = log minimum angular resolution visual acuity, IOP = intraocular pressure, PRP = panretinal photocoagulation, PPV = pars plana vitrectomy, DCPC = diode laser cyclophotocoagulation, TLE = trabeculectomy. * Mean ± SD. ^†^ Median, range. ^§^ Number (%).

**Table 2:** Surgical procedures and the numbers performed for diabetic retinopathy eyes with neovascular glaucoma.

	Number (%)
PPV–PRCP	24 (77.4)
0	7 (22.6)
1	9 (29.0)
2	10 (32.2)
3	3 (9.7)
4	2 (6.5)
Average number of times per eye	1.5 ± 1.2
DCPC	11 (35.5)
0	20 (64.5)
1	11 (35.5)
Average number of times per eye	0.4 ± 0.5
IVB	13 (41.9)
0	18 (58.1)
1	11 (35.5)
2	2 (6.5)
Average number of times per eye	0.5 ± 0.6
TLE–MMC	27 (87.1)
0	4 (12.9)
1	22 (71.0)
2	2 (6.5)
3	2 (6.5)
4	1 (3.2)
Average number of times per eye	1.2 ± 0.9

PPV = pars plana vitrectomy, PRCP = panretinal and ciliary photocoagulation, DCPC = diode laser cyclophotocoagulation, IVB = intravitreal bevacizumab injection, TLE = trabeculectomy, MMC = mitomycin C.

**Table 3:** Comparison of neovascular glaucoma patients who received trabeculectomy with mitomycin C between procedures, with and without prior bevacizumab injection.

	IVB (+) (n = 13)	IVB (−) (n = 14)	*p* Value
Age (years)	62.6 ± 10.4 *	67.1 ± 12.9	0.2536
Male/Female	9/4	10/4	0.9006
Follow-up periods (weeks)	29.4 ± 34.1	29.4 ± 31.0	0.8841
The initial presentation			
LogMAR VA	1.28 ± 1.01 (1.05, 0–2.89) ^†^	1.34 ± 1.10 (1.20, 0–2.89)	0.9418
IOP	35.2 ± 12.1 (31.0, 21–60)	33.9 ± 18.8 (27.5, 16.5–78)	0.3438
PRP	12 (92.3) ^§^	11 (78.6)	0.3154
Cataract surgery	10 (76.9)	11 (78.6)	0.9180
PPV	9 (69.2)	9 (64.3)	0.7854
FBS (mg/dL)	145.7 ± 28.1	152.4 ± 47.4	0.7709
HbA1c (%)	7.2 ± 1.1	7.9 ± 1.8	0.2637
Creatinine (mg/dL)	2.6 ± 3.5	2.0 ± 3.1	0.9806
BUN (mg/dL)	30.1 ± 15.5	24.5 ± 19.4	0.1234
eGFR (mL/min/1.73 m^2^)	56.7 ± 30.9	53.3 ± 27.1	0.6799
The last presentation			
LogMAR VA	1.44 ± 1.06 (1.05, 0–2.89)	1.19 ± 1.06 (0.85, −0.08–2.89)	0.5110
IOP (mmHg)	11.4 ± 4.8 (11, 3–21)	15.9 ± 9.1 (16, 3–42.3)	0.0613
Cataract surgery	13 (100)	14 (100)	1
PPV with PRCP	1.7 ± 1.2	1.6 ± 1.2	0.8204
DCPC	0.31 ± 0.48	0.37 ± 0.50	0.7266
TLE + MMC	1.31 ± 0.85	1.0 ± 0.88	0.1978

IVB = intravitreal bevacizumab injection (IVB), LogMAR VA = log minimum angular resolution visual acuity, IOP = intraocular pressure, PRP = panretinal photocoagulation, PPV = pars plana vitrectomy, FBS = fasting blood sugar, BUN = blood urea nitrogen, eGFR = estimated glomerular filtration rate, PRCP = panretinal and ciliary photocoagulation, DCPC = diode laser cyclophotocoagulation, TLE = trabeculectomy, MMC = mitomycin C. * Mean ± SD. ^†^ Median, range. Number (%). ^§^ Number (%).

**Table 4:** Comparison between neovascular glaucoma patients with LogMAR VA greater than 1 and those with LogMAR VA of 1 or less at the initial visit.

	LogMAR VA > 1 (n = 15)	LogMAR VA ≤ 1 (n = 16)	*p* Value
Age (years)	62.5 ± 12.8 *	65.8 ± 11.4	0.4642
Male/Female	8/7	14/2	0.0362
Follow-up periods (weeks)	34.0 ± 32.9	30.0 ± 33.4	0.6779
The initial presentation			
IOP	40.5 ± 18.4 (38.0, 21–78) ^†^	26.0 ± 5.9 (24.3, 16.5–38)	0.0167
PRP	11 (73.3) ^§^	14 (87.5)	0.3184
Cataract surgery	10 (66.7)	13 (81.3)	0.3538
PPV	8 (53.3)	10 (62.5)	0.6052
FBS (mg/dL)	159.0 ± 40.0	137.6 ± 35.8	0.1989
HbA1c (%)	7.6 ± 1.7	7.2 ± 1.2	0.8123
Creatinine (mg/dL)	1.7 ± 1.6	2.6 ± 3.9	0.2767
BUN (mg/dL)	23.5 ± 13.9	29.9 ± 18.9	0.2992
eGFR (mL/min/1.73 m^2^)	60.9 ± 30.0	49.7 ± 25.1	0.3529
The last presentation			
LogMAR VA	1.86 ± 1.01 (2, 0.15–2.89)	0.70 ± 0.82 (0.61, −0.18–2.89)	0.0026
IOP (mmHg)	12.4 ± 6.6 (13.0, 3–22)	15.5 ± 8.0 (14.6, 6–42.3)	0.4884
IVB	0.6 ± 0.7	0.4 ± 0.5	0.4544
Cataract surgery	15 (100)	16 (100)	1
PPV with PRCP	1.6 ± 1.3	1.4 ± 1.0	0.6084
DCPC	5 (33.3)	6 (37.5)	0.8085
TLE + MMC	1.3 ± 1.0	1.1 ± 0.8	0.6746

LogMAR VA = log minimum angular resolution visual acuity, IOP = intraocular pressure, PRP = panretinal photocoagulation, PPV = pars plana vitrectomy, FBS = fasting blood sugar, BUN = blood urea nitrogen, eGFR = estimated glomerular filtration rate, IVB = intravitreal bevacizumab injection (IVB), PRCP = panretinal and ciliary photocoagulation, DCPC = diode laser cyclophotocoagulation, TLE = trabeculectomy, MMC = mitomycin C. * Mean ± SD. ^†^ Median, range. Number (%). ^§^ Number (%).

**Table 5:** Comparison between neovascular glaucoma patients with IOP of 30 mmHg or less and those with IOP higher than 30 mmHg at the initial visit.

	IOP > 30 mmHg (n = 13)	IOP ≤ 30 mmHg (n = 18)	*p* Value
Age (years)	59.7 ± 12.0 *	67.4 ± 11.1	0.0624
Male/Female	9/4	13/5	0.8563
Follow-up periods (weeks)	36.8 ± 35.6	28.5 ± 31.3	0.5613
The initial presentation			
LogMAR VA	1.62 ± 1.04 (1.52, 0.10–2.89) ^†^	1.03 ± 1.02 (0.85, 0–2.89)	0.0840
IOP (mmHg)	46.2 ± 15.4 (42.0, 30.7–78)	23.5 ± 3.5 (23.2, 16.5–29.3)	<0.0001
PRP	9 (69.2) ^§^	16 (88.9)	0.1716
Cataract surgery	9 (69.2)	14 (77.8)	0.5915
PPV	9 (69.2)	9 (50.0)	0.2843
FBS (mg/dL)	145.3 ± 37.3	149.8 ± 40.8	0.7793
HbA1c (%)	7.3 ± 1.1	7.5 ± 1.7	0.9202
Creatinine (mg/dL)	3.1 ± 4.4	1.4 ± 1.3	0.6887
BUN (mg/dL)	31.7 ± 22.0	22.9 ± 10.1	0.3713
eGFR (mL/min/1.73 m^2^)	52.9 ± 34.8	56.7 ± 22.2	0.9697
The last presentation			
LogMAR VA	1.81 ± 1.03 (2.0, 0.52–2.89)	0.87 ± 0.96 (0.76, −0.18–2.89)	0.0286
IOP (mmHg)	12.4 ± 5.6 (12.0, 3–21)	15.2 ± 8.4 (14.6, 4–42.3)	0.4343
IVB	0.8 ± 0.7	0.3 ± 0.5	0.0410
Cataract surgery	13 (100)	18 (100)	1
PPV with PRCP	1.9 ± 1.3	1.2 ± 0.9	0.1055
TLE + MMC	1.4 ± 1.0	1.0 ± 0.8	0.2500
DCPC	4 (30.8)	7 (38.9)	0.6410

LogMAR VA = log minimum angular resolution visual acuity, IOP = intraocular pressure, PRP = panretinal photocoagulation, PPV = pars plana vitrectomy, FBS = fasting blood sugar, BUN = blood urea nitrogen, eGFR = estimated glomerular filtration rate, IVB = intravitreal bevacizumab injection (IVB), PRCP = panretinal and ciliary photocoagulation, DCPC = diode laser cyclophotocoagulation, TLE = trabeculectomy, MMC = mitomycin C. * Mean ± SD. ^†^ Median, range. Number (%). ^§^ Number (%).

## Data Availability

The research data supporting the findings of this study are available from the corresponding author upon reasonable request.
